# 3-Eth­oxy-2-(1,3-thia­zol-2-yl)isoindolin-1-one

**DOI:** 10.1107/S1600536809038884

**Published:** 2009-09-30

**Authors:** Wenkuan Li, Handong Yin, Liyuan Wen, Jichun Cui, Daqi Wang

**Affiliations:** a College of Chemistry and Chemical Engineering, Liaocheng University, Shandong 252059, People’s Republic of China

## Abstract

In the title compound, C_13_H_12_N_2_O_2_S, the dihedral angles between the isoindolone ring system and the thia­zole ring and the eth­oxy group are 6.50 (11) and 89.0 (2)°, respectively.

## Related literature

For general background to isoindolin-1-one derivatives, see: Gai *et al.* (2003 ▶). For hybridization, see: Beddoes *et al.* (1986 ▶).
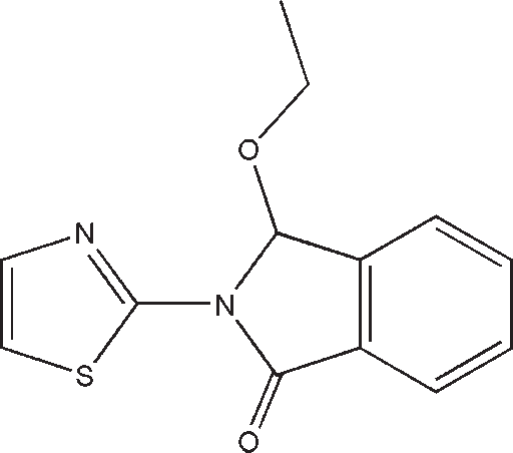


## Experimental

### 

#### Crystal data

C_13_H_12_N_2_O_2_S
*M* *_r_* = 260.31
Monoclinic, 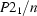

*a* = 8.0933 (11) Å
*b* = 9.1406 (14) Å
*c* = 17.2077 (19) Å
β = 98.720 (1)°
*V* = 1258.3 (3) Å^3^
*Z* = 4
Mo *K*α radiation
μ = 0.25 mm^−1^
*T* = 298 K
0.50 × 0.49 × 0.47 mm

#### Data collection

Siemens SMART CCD area-detector diffractometer
Absorption correction: multi-scan (*SADABS*; Sheldrick, 1996 ▶) *T*_min_ = 0.884, *T*_max_ = 0.891
6305 measured reflections
2236 independent reflections
1554 reflections with *I* > 2σ(*I*)
*R*_int_ = 0.031

#### Refinement

*R*[*F* ^2^ > 2σ(*F* ^2^)] = 0.042
*wR*(*F* ^2^) = 0.117
*S* = 1.04
2236 reflections
164 parameters
H-atom parameters constrained
Δρ_max_ = 0.20 e Å^−3^
Δρ_min_ = −0.24 e Å^−3^

### 

Data collection: *SMART* (Siemens, 1996 ▶); cell refinement: *SAINT* (Siemens, 1996 ▶); data reduction: *SAINT*; program(s) used to solve structure: *SHELXS97* (Sheldrick, 2008 ▶); program(s) used to refine structure: *SHELXL97* (Sheldrick, 2008 ▶); molecular graphics: *SHELXTL* (Sheldrick, 2008 ▶); software used to prepare material for publication: *SHELXTL*.

## Supplementary Material

Crystal structure: contains datablocks I, global. DOI: 10.1107/S1600536809038884/bx2243sup1.cif

Structure factors: contains datablocks I. DOI: 10.1107/S1600536809038884/bx2243Isup2.hkl

Additional supplementary materials:  crystallographic information; 3D view; checkCIF report

## Figures and Tables

**Fig. 1.**
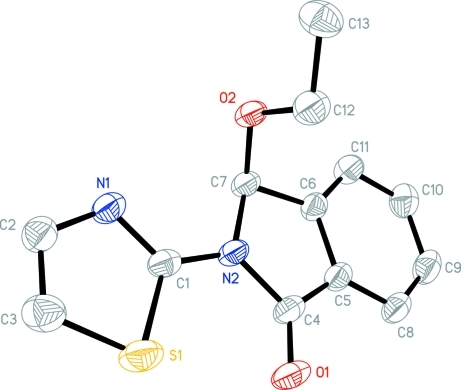
The molecular structure of the compound, showing 50% probability displacement ellipsoids. H atoms have been omitted for clarity.

## supplementary crystallographic information

### Comment

The title compound, (I), was formed by accident, instead of the organotin compound containing schiff base which was expected. Isoindolin-1-one derivatives have been demonstrated to possess anxiolytic activity and are of interest as sedatives, hypnotics and muscle relaxants. In addition,the isoindolone moiety also features in anti-cancer drug candidates including protein kinase inhibitors(Gai *et al.* 2003). We have synthesized the title compound(I) and its crystal structure is reported herein.The molecular structure of (I) is shown in Fig.1. The dihedral angles between the isoindolone ring system and thiazole ring and oxyethyl group are 6.50 (11) and 89.0 (2)° respectively.The sum of bond angles around atom N1(359.6 °) indicates that the atom N1 is in *sp*^2^ hybridized state (Beddoes *et al.* 1986). The crystal packing is stabilized mainly by van der Waals interactions.

### Experimental

(*E*)-2-((thiazol-2-ylimino)methyl)benzoic acid (4 mmol) and sodium ethoxide (4 mmol) were added to a stirred solution of ethanol (30 ml) in a Schlenk flask and stirred for 0.5 h. Chlorotriphenyltin (4 mmol) was then added to the reactor and the reaction mixture was heated under reflux for 6 h. The resulting clear solution was evaporated under vacuum. The product was crystallized from a mixed of dichloromethane/petroleum (1:1) to afford the title compound unexpectedly. Anal. Calcd (%) for C_13_H_12_N_2_O_2_S (Mr = 260.31): C, 59.98; H, 4.65; N, 10.76; O, 12.29; S, 12.32 Found (%): C, 60.00; H, 4.62; N, 10.74; O, 12.31; S, 12.30

### Refinement

H atoms were positioned geometrically, with C—H = 0.93, 0.96, 0.97 and 0.98 Å for aromatic, methyl, methylene and methine H atoms, respectively, and constrained to ride on their parent atoms, with *U*_iso_(H) = *x U*_eq_(C) where *x* =1.5 for methyl H and *x* = 1.2 for all other H atoms.

### Crystal data

**Table:**

C_13_H_12_N_2_O_2_S	*F*(000) = 544
*M**_r_* = 260.31	*D*_x_ = 1.374 Mg m^−^^3^
Monoclinic, *P*2_1_/*n*	Mo *K*α radiation, λ = 0.71073 Å
Hall symbol: -P 2yn	Cell parameters from 2091 reflections
*a* = 8.0933 (11) Å	θ = 2.5–26.6°
*b* = 9.1406 (14) Å	µ = 0.25 mm^−^^1^
*c* = 17.2077 (19) Å	*T* = 298 K
β = 98.720 (1)°	Block, colourless
*V* = 1258.3 (3) Å^3^	0.50 × 0.49 × 0.47 mm
*Z* = 4	

### Data collection

**Table:**

Siemens SMART CCD area-detector diffractometer	2236 independent reflections
Radiation source: fine-focus sealed tube	1554 reflections with *I* > 2σ(*I*)
graphite	*R*_int_ = 0.031
φ and ω scans	θ_max_ = 25.1°, θ_min_ = 2.4°
Absorption correction: multi-scan (*SADABS*; Sheldrick, 1996)	*h* = −9→9
*T*_min_ = 0.884, *T*_max_ = 0.891	*k* = −10→8
6305 measured reflections	*l* = −20→18

### Refinement

**Table:**

Refinement on *F*^2^	Primary atom site location: structure-invariant direct methods
Least-squares matrix: full	Secondary atom site location: difference Fourier map
*R*[*F*^2^ > 2σ(*F*^2^)] = 0.042	Hydrogen site location: inferred from neighbouring sites
*wR*(*F*^2^) = 0.117	H-atom parameters constrained
*S* = 1.04	*w* = 1/[σ^2^(*F*_o_^2^) + (0.0456*P*)^2^ + 0.5551*P*] where *P* = (*F*_o_^2^ + 2*F*_c_^2^)/3
2236 reflections	(Δ/σ)_max_ < 0.001
164 parameters	Δρ_max_ = 0.20 e Å^−^^3^
0 restraints	Δρ_min_ = −0.24 e Å^−^^3^

### Fractional atomic coordinates and isotropic or equivalent isotropic displacement parameters (Å^2^)

**Table:**

	*x*	*y*	*z*	*U*_iso_*/*U*_eq_	
S1	0.40181 (10)	0.62694 (9)	0.23706 (4)	0.0734 (3)	
N1	0.5832 (2)	0.5184 (2)	0.14152 (12)	0.0558 (6)	
N2	0.3056 (2)	0.4365 (2)	0.11652 (11)	0.0482 (5)	
O1	0.0910 (2)	0.5044 (2)	0.18270 (11)	0.0663 (5)	
O2	0.44903 (19)	0.23876 (18)	0.06204 (9)	0.0515 (4)	
C1	0.4324 (3)	0.5174 (3)	0.15857 (14)	0.0479 (6)	
C2	0.6825 (3)	0.6082 (3)	0.19276 (15)	0.0603 (7)	
H2	0.7953	0.6217	0.1899	0.072*	
C3	0.6079 (4)	0.6747 (4)	0.24650 (17)	0.0713 (8)	
H3	0.6608	0.7387	0.2843	0.086*	
C4	0.1397 (3)	0.4400 (3)	0.12897 (15)	0.0517 (6)	
C5	0.0457 (3)	0.3538 (3)	0.06545 (14)	0.0491 (6)	
C6	0.1552 (3)	0.3004 (3)	0.01772 (14)	0.0484 (6)	
C7	0.3314 (3)	0.3506 (3)	0.04657 (13)	0.0463 (6)	
H7	0.3668	0.4169	0.0075	0.056*	
C8	−0.1242 (3)	0.3255 (3)	0.04990 (16)	0.0580 (7)	
H8	−0.1971	0.3625	0.0819	0.070*	
C9	−0.1811 (3)	0.2406 (3)	−0.01463 (18)	0.0677 (8)	
H9	−0.2947	0.2200	−0.0265	0.081*	
C10	−0.0725 (4)	0.1850 (3)	−0.06238 (18)	0.0689 (8)	
H10	−0.1140	0.1277	−0.1056	0.083*	
C11	0.0976 (3)	0.2142 (3)	−0.04631 (16)	0.0605 (7)	
H11	0.1708	0.1764	−0.0780	0.073*	
C12	0.4196 (4)	0.1366 (3)	0.12151 (17)	0.0682 (8)	
H12A	0.3049	0.1023	0.1115	0.082*	
H12B	0.4381	0.1833	0.1727	0.082*	
C13	0.5357 (4)	0.0121 (3)	0.12016 (18)	0.0747 (9)	
H13A	0.5135	−0.0360	0.0701	0.112*	
H13B	0.5203	−0.0559	0.1610	0.112*	
H13C	0.6488	0.0473	0.1286	0.112*	

### Atomic displacement parameters (Å^2^)

**Table:**

	*U*^11^	*U*^22^	*U*^33^	*U*^12^	*U*^13^	*U*^23^
S1	0.0675 (5)	0.0909 (6)	0.0664 (5)	0.0092 (4)	0.0252 (4)	−0.0195 (4)
N1	0.0455 (12)	0.0655 (14)	0.0595 (13)	0.0005 (10)	0.0185 (10)	−0.0066 (11)
N2	0.0414 (11)	0.0518 (12)	0.0558 (12)	0.0053 (9)	0.0216 (9)	0.0009 (10)
O1	0.0562 (11)	0.0790 (13)	0.0707 (12)	0.0133 (10)	0.0320 (9)	−0.0043 (10)
O2	0.0436 (9)	0.0534 (10)	0.0618 (10)	0.0085 (8)	0.0218 (8)	0.0015 (8)
C1	0.0495 (14)	0.0485 (14)	0.0489 (13)	0.0101 (11)	0.0179 (11)	0.0071 (11)
C2	0.0508 (15)	0.0687 (18)	0.0615 (16)	−0.0015 (13)	0.0088 (13)	−0.0059 (14)
C3	0.0709 (19)	0.082 (2)	0.0600 (17)	0.0074 (16)	0.0064 (14)	−0.0130 (16)
C4	0.0445 (14)	0.0542 (15)	0.0612 (15)	0.0114 (12)	0.0237 (12)	0.0133 (13)
C5	0.0424 (13)	0.0491 (14)	0.0590 (15)	0.0065 (11)	0.0177 (11)	0.0150 (12)
C6	0.0435 (14)	0.0473 (14)	0.0570 (15)	0.0023 (11)	0.0164 (11)	0.0109 (12)
C7	0.0408 (13)	0.0480 (14)	0.0536 (14)	0.0065 (11)	0.0183 (11)	0.0053 (11)
C8	0.0441 (15)	0.0623 (17)	0.0712 (18)	0.0083 (13)	0.0200 (13)	0.0185 (15)
C9	0.0441 (15)	0.076 (2)	0.084 (2)	−0.0033 (14)	0.0124 (14)	0.0152 (17)
C10	0.0607 (18)	0.0698 (19)	0.0747 (19)	−0.0109 (15)	0.0059 (14)	−0.0009 (16)
C11	0.0529 (16)	0.0644 (17)	0.0672 (17)	0.0014 (13)	0.0186 (13)	0.0014 (15)
C12	0.0670 (18)	0.0639 (18)	0.0779 (19)	0.0119 (14)	0.0242 (15)	0.0180 (15)
C13	0.078 (2)	0.0633 (19)	0.078 (2)	0.0156 (16)	−0.0028 (16)	0.0035 (16)

### Geometric parameters (Å, °)

**Table:**

S1—C3	1.708 (3)	C6—C11	1.377 (4)
S1—C1	1.729 (2)	C6—C7	1.509 (3)
N1—C1	1.298 (3)	C7—H7	0.9800
N1—C2	1.372 (3)	C8—C9	1.376 (4)
N2—C1	1.378 (3)	C8—H8	0.9300
N2—C4	1.392 (3)	C9—C10	1.388 (4)
N2—C7	1.478 (3)	C9—H9	0.9300
O1—C4	1.211 (3)	C10—C11	1.388 (4)
O2—C7	1.395 (3)	C10—H10	0.9300
O2—C12	1.432 (3)	C11—H11	0.9300
C2—C3	1.326 (4)	C12—C13	1.479 (4)
C2—H2	0.9300	C12—H12A	0.9700
C3—H3	0.9300	C12—H12B	0.9700
C4—C5	1.464 (4)	C13—H13A	0.9600
C5—C6	1.385 (3)	C13—H13B	0.9600
C5—C8	1.385 (3)	C13—H13C	0.9600
			
C3—S1—C1	88.22 (13)	O2—C7—H7	108.8
C1—N1—C2	109.8 (2)	N2—C7—H7	108.8
C1—N2—C4	124.5 (2)	C6—C7—H7	108.8
C1—N2—C7	121.80 (17)	C9—C8—C5	117.6 (2)
C4—N2—C7	113.3 (2)	C9—C8—H8	121.2
C7—O2—C12	115.58 (17)	C5—C8—H8	121.2
N1—C1—N2	122.4 (2)	C8—C9—C10	121.3 (3)
N1—C1—S1	115.1 (2)	C8—C9—H9	119.4
N2—C1—S1	122.52 (17)	C10—C9—H9	119.4
C3—C2—N1	116.0 (3)	C11—C10—C9	120.7 (3)
C3—C2—H2	122.0	C11—C10—H10	119.7
N1—C2—H2	122.0	C9—C10—H10	119.7
C2—C3—S1	111.0 (2)	C6—C11—C10	118.3 (2)
C2—C3—H3	124.5	C6—C11—H11	120.8
S1—C3—H3	124.5	C10—C11—H11	120.8
O1—C4—N2	124.2 (3)	O2—C12—C13	108.4 (2)
O1—C4—C5	129.8 (2)	O2—C12—H12A	110.0
N2—C4—C5	106.1 (2)	C13—C12—H12A	110.0
C6—C5—C8	121.7 (3)	O2—C12—H12B	110.0
C6—C5—C4	109.0 (2)	C13—C12—H12B	110.0
C8—C5—C4	129.3 (2)	H12A—C12—H12B	108.4
C11—C6—C5	120.4 (2)	C12—C13—H13A	109.5
C11—C6—C7	128.8 (2)	C12—C13—H13B	109.5
C5—C6—C7	110.8 (2)	H13A—C13—H13B	109.5
O2—C7—N2	114.23 (19)	C12—C13—H13C	109.5
O2—C7—C6	115.0 (2)	H13A—C13—H13C	109.5
N2—C7—C6	100.76 (17)	H13B—C13—H13C	109.5
